# Coagulotoxic Cobras: Clinical Implications of Strong Anticoagulant Actions of African Spitting *Naja* Venoms That Are Not Neutralised by Antivenom but Are by LY315920 (Varespladib)

**DOI:** 10.3390/toxins10120516

**Published:** 2018-12-04

**Authors:** Mátyás A. Bittenbinder, Christina N. Zdenek, Bianca op den Brouw, Nicholas J. Youngman, James S. Dobson, Arno Naude, Freek J. Vonk, Bryan G. Fry

**Affiliations:** 1 Venom Evolution Lab, School of Biological Sciences, University of Queensland, St Lucia, QLD 4072 Australia; mbittenbinder@gmail.com (M.A.B.); christinazdenek@gmail.com (C.N.Z.); bianca_odb@hotmail.com (B.o.d.B.); nicholas.youngman@uq.net.au (N.J.Y.); j.dobson@uq.edu.au (J.S.D.); 2 Institute of Biology, Leiden University, 2300 RA Leiden, The Netherlands; 3 Snakebite Assist, Pretoria ZA-0001, South Africa; afnaude@worldonline.co.za; 4 Naturalis Biodiversity Center, 2333 CR Leiden, The Netherlands

**Keywords:** cobra, venom, antivenom, snakebite, coagulopathy, coagulotoxicity, tissue damage, varespladib, LY315920

## Abstract

Snakebite is a global tropical disease that has long had huge implications for human health and well-being. Despite its long-standing medical importance, it has been the most neglected of tropical diseases. Reflective of this is that many aspects of the pathology have been underinvestigated. Snakebite by species in the Elapidae family is typically characterised by neurotoxic effects that result in flaccid paralysis. Thus, while clinically significant disturbances to the coagulation cascade have been reported, the bulk of the research to date has focused upon neurotoxins. In order to fill the knowledge gap regarding the coagulotoxic effects of elapid snake venoms, we screened 30 African and Asian venoms across eight genera using in vitro anticoagulant assays to determine the relative inhibition of the coagulation function of thrombin and the inhibition of the formation of the prothrombinase complex through competitive binding to a nonenzymatic site on Factor Xa (FXa), thereby preventing FXa from binding to Factor Va (FVa). It was revealed that African spitting cobras were the only species that were potent inhibitors of either clotting factor, but with Factor Xa inhibited at 12 times the levels of thrombin inhibition. This is consistent with at least one death on record due to hemorrhage following African spitting cobra envenomation. To determine the efficacy of antivenom in neutralising the anticoagulant venom effects, for the African spitting cobras we repeated the same 8-point dilution series with the addition of antivenom and observed the shift in the area under the curve, which revealed that the antivenom performed extremely poorly against the coagulotoxic venom effects of all species. However, additional tests with the phospholipase A_2_ inhibitor LY315920 (trade name: varespladib) demonstrated a powerful neutralisation action against the coagulotoxic actions of the African spitting cobra venoms. Our research has important implications for the clinical treatment of cobra snakebites and also sheds light on the molecular mechanisms involved in coagulotoxicity within *Naja*. As the most coagulotoxic species are also those that produce characteristic extreme local tissue damage, future research should investigate potential synergistic actions between anticoagulant toxins and cytotoxins.

## 1. Introduction

Envenomation as a result of snakebites is an important public health issue, mainly in the tropical and subtropical countries of the developing world. A total of 5.8 billion people are within a short distance from venomous snakes, or directly within the habitat range, with estimates ranging between 1.8 million and 5.4 million snakebite victims annually, and mortality rates surpassing 100,000 individuals [1,2,3,4,5]. Snake venoms can produce a range of local and systemic effects in humans, with some being life-threatening and others being permanently debilitating. Importantly, without early and effective antivenom treatment, morbidity following snakebite can cause permanent disability and/or disfigurement and can severely inhibit one’s ability to work [6]. As most snakebite cases occur in developing countries in sub-Saharan Africa, India, Southeast Asia, and Latin America and are likely to be male agricultural workers, the socio-economic impact of snakebite on families and local economies can be major [7]. Exacerbating the snakebite issue is a longstanding lack of attention and funding from governments, healthcare authorities, and pharmaceutical companies. Due to this persistent problem and global neglect, the World Health Organization (WHO) recently included snakebite in the list of globally neglected tropical diseases and started initiatives to raise global awareness [1].

The most life-threatening pathology in elapid snakebite victims derives from neurotoxins, which can cause paralysis of the diaphragm and eventually asphyxiation [6]. As such, antivenom efficacy research with elapid venoms to date has focused upon the neutralisation of neurotoxins [8]. Due to neurotoxicity being responsible for the lethal clinical effects, other potential pathological actions of snake venoms have been comparably neglected, with anticoagulation being particularly data deficient. Venom effects on the coagulation cascade that could cause anticoagulant effects include inhibition of the blood coagulation cascade enzymes thrombin and Factor Xa (FXa) [9]. Several cobra species have been shown to produce anticoagulant effects by inhibiting blood coagulation factors through the use of modified Group I phosopholipase A_2_ (PLA_2_) toxins. For example, inhibitors of the enzymatic activities of FXa and thrombin have been isolated from the non-spitting African cobra *N. haje* [10] and the non-spitting Asian cobra *N. kaouthia* [11]. Novel FXa inhibitors which do not block the FXa enzymatic site but bind to FXa interfere with FXa’s ability to bind to Factor Va (FVa)—and thereby form the prothrombinase complex—have been isolated from the venom of the African spitting cobra *N. nigricollis* [12,13,14]. In all of these reports, the work was undertaken on purified toxins, which are in low amounts in *N. haje* and *N. kaouthia*. Therefore, the relative anticoagulant potency of the crude venom, which is what would be seen by evolutionary selection pressures, is unknown; consequently, the phylogenetic patterns have not been elucidated.

In most envenomations by snakes, antivenoms are the only effective medical treatment, but problems exist with antivenom shortages, ineffective antivenoms, or delays in reaching care rendering otherwise good treatments ineffective [2]. For example, previous studies have shown that localised effects such as haemorrhage or tissue damage can be poorly neutralised by available antivenoms [6].

We set out to fill the aforementioned knowledge gap by performing in vitro functional activity tests on a wide range of African and Asian elapid venoms to determine their relative coagulotoxicity, as well as the precise mechanism of action on the blood coagulation cascade. Additionally, we directly compare the efficacy of both the South African polyvalent antivenom (SAIMR) and LY315920 (trade name varespladib), a promising candidate for broad-spectrum inhibition of snakebite toxicity mediated by PLA_2_, against the coagulotoxic effects of these venoms. Our results not only demonstrate evolutionary patterns underlying anticoagulant activity within elapid species but also have implications for the clinical treatment of elapid snakebites.

## 2. Results

**Figure 1:**
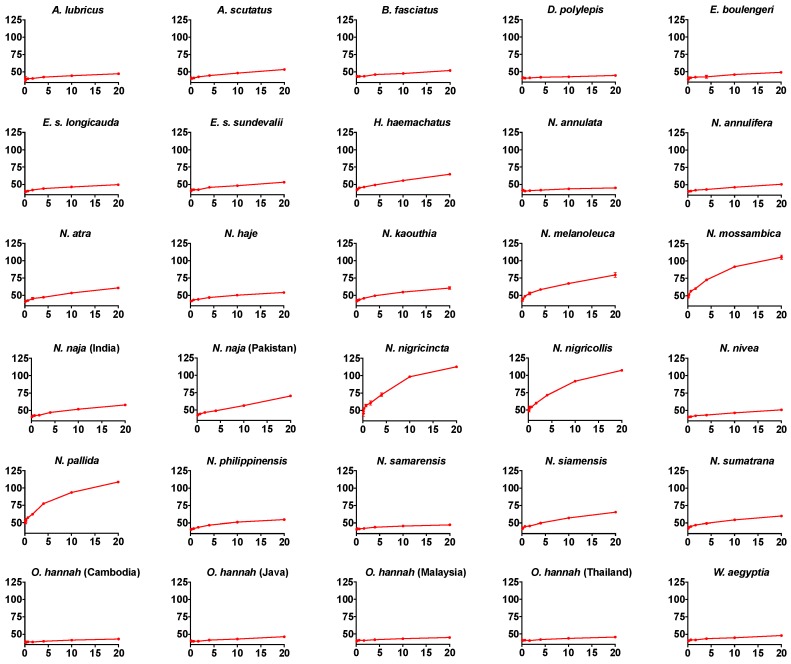
Dose–response curves for thrombin inhibition. A comparison of clotting curves showing the relative inhibitory effects of the venom of 30 different species of elapid snakes on thrombin. *x* axis: venom concentration (µg/mL); *y* axis: clotting time in seconds. Negative control values were 43.4 ± 0.6 s. Data points are mean and standard deviations for *N* = 3. Note that for most data points, the error bars are smaller than the line icon. Note also that the *y* axis begins at 35 to improve viewability.

**Figure 2:**
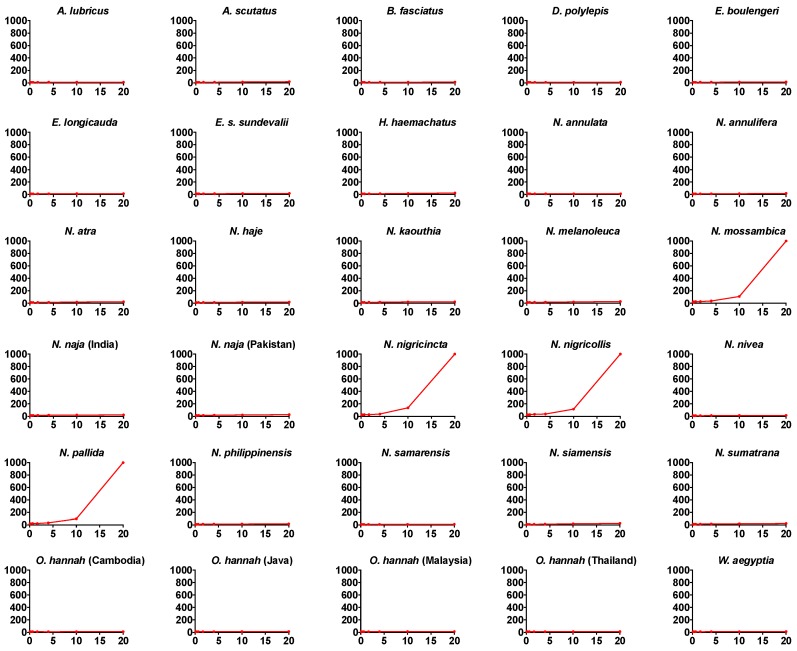
Dose–response curves for Factor Xa (FXa) inhibition. A comparison of clotting curves showing the relative inhibitory effects of the venom of 30 different species of elapid snakes on FXa. *x* axis: venom concentration (µg/mL); *y* axis: clotting time in seconds, with a machine maximum reading time of 999 s. Negative control values were 11.9 ± 0.1 s. Data points are mean and standard deviations for *N* = 3. Note that for most data points, the error bars are smaller than the line icon.

**Figure 3:**
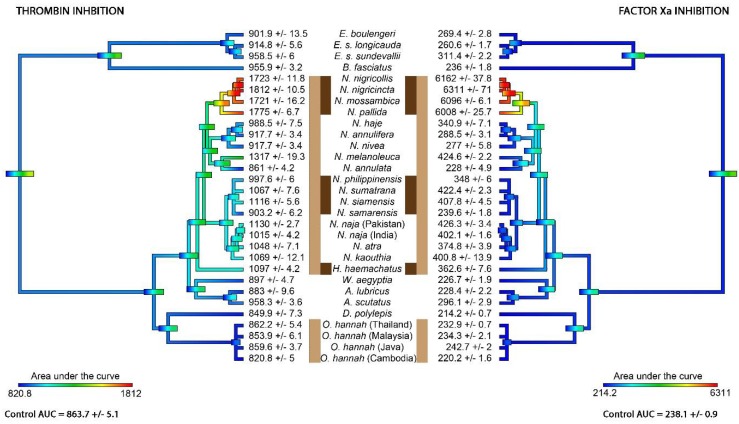
Ancestral state reconstruction of thrombin and FXa inhibition. A reconstruction of the ancestral state of thrombin and FXa inhibition, based on AUC (area under the curve) values derived from dose–response curves for thrombin and FXa inhibition by venoms. Warmer colours represent larger AUCs (greater anticoagulant venom potency). The two clades which have independently evolved hooding defensive displays are indicated with light brown vertical bards, while the three lineages which have evolved defensive spitting are indicated with dark brown vertical bars [16]. Phylogeny based upon [17,18].

A series of anticoagulant assays were performed on all venoms, including 8-point dose–response curves, to investigate the inhibition of thrombin’s ability to clot human fibrinogen (Figure 1) and on the impediment of Factor Xa’s ability to clot recalcified plasma by forming a prothrombinase complex with Factor Va (Figure 2). Only the African spitting *Naja* species significantly inhibited thrombin, and these species were the most potent in blocking Factor Xa’s ability to form a prothrombinase complex with Factor Va. These venoms did not significantly interfere with FXa’s enzymatic activity upon a colormetric substrate (e.g., negative control of 1.686 ± 0.0226 absorbance units versus 1.575 ± 0.0475 for *N. nigricollis*), but instead competed with Factor Va for binding to FXa, as has been previously described for isolated *N. nigricollis* PLA_2_ [12,13,14]. Therefore, this unique mode of activity would be missed by conventional assay setups that simply test for ability to interfere with FXa’s enzymatic activity (c.f. [15]). Other species within the *Hemachatus/Naja* clade displayed much lower levels of activity for both activities. The four species within the clade of African spitting cobras (*N. mossambica*, *N. nigricincta*, *N. nigricollis*, and *N. pallida*) exhibited 12 times greater an inhibition effect on FXa (25 times the negative control) compared to that on thrombin (2 times the negative control). However, there was an extremely strong linkage between the two toxic activities (PGLS: *t* = 7.1253, *p* = 0.00000001, df = 1) with the most potent species on one being the most potent species on the other. A phylogenetic ancestral state reconstruction of venom effects revealed that thrombin and FXa inhibition activities both evolved at the base of the *Hemachatus*/*Naja* clade, but both were only amplified in the African spitting *Naja* (Figure 3).

**Figure 4:**
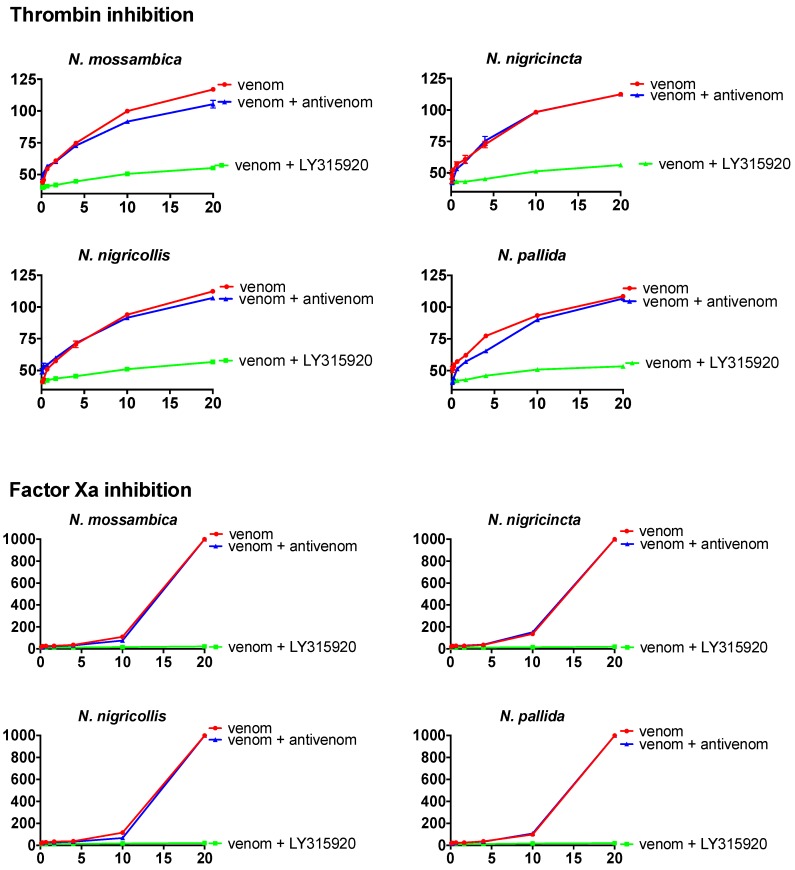
Dose–response curves for LY315920 (varespladib) and SAIMR antivenom efficacy. Comparison of clotting curves showing the relative inhibitory effects of venom of the four most potent anticoagulant African spitting *Naja* species, with and without the addition of antivenom or LY315920. *x* axis: venom concentration (µg/mL); *y* axis: clotting time in seconds. Thrombin negative control values were 43.4 ± 0.6 s. FXa negative control values were 11.9 ± 0.1 s. Data points are mean and standard deviations for *N* = 3. Note that for most data points, the error bars are smaller than the line icon. Also, the venom + antivenom blue line is not visible in some doses/species due to the lack of effect resulting in an identical line to that of the venom-only red line. To improve viewability, the *y* axis of thrombin inhibition begins at 35.

Consistent with clinical observations of antivenom ineffectiveness [19], the antivenom efficacy testing in this study revealed an inability of the South African Institute for Medical Research antivenom to neutralise both FXa and thrombin inhibition activity by venoms (Figure 4). Even *N. mossambica*, one of the immunising venoms, was only negligibly neutralised for thrombin inhibition activity and not at all for FXa inhibition activity. All other species were virtually untouched for thrombin and not at all for FXa. However, testing of LY315920 (trade name: varespladib) at the same concentration previously shown to be effective for other types of venom toxicity [20] was shown in this study to possess a strong ability to neutralise both thrombin and FXa inhibition activity by venoms (Figure 4).

## 3. Discussion

This study revealed for the first time that thrombin inhibition and inhibition of prothrombinase complex formation by competitive binding to FXa, thus preventing FVa’s binding to FXa, are shared features of the *Hemachatus*/*Naja* clade but are potent only in the African spitting *Naja* species, with FXa inhibition more strongly amplified than thrombin inhibition (Figure 3). While the prothrombinase complex formation inhibition activity was 12 times stronger than that of the thrombin inhibition activity, the inhibition of thrombin would exhibit synergistic effects in combination with FXa inhibition due to the fact that the two enzymes are linked in the clotting cascade and would otherwise produce positive feedback loops that result in more clots. Local and systemic coagulation disturbances have been observed in victims envenomated by the African spitting cobra species *Naja nigricincta*, [21], and laboratory tests on *N. nigricincta* have confirmed the potenty ability to produce haemorrhage [22]. These haemorrhagic clinical observations and laboratory results are consistent with the anticoagulation results in this study. Similarly, haemorrhage has been reported for *N. nigricollis* envenomations and attributed as the cause of death in at least one case [19]. In contrast, case study reports of non-spitting *Naja* species do not report haemorrhagic effects in bite victims [6,23]. Consistent with this dichotomy in clinical effects, while the clade of African spitting cobras exhibited potent anticoagulant venom effects, the same was not observed within other spitting cobras in the two other clades (*Hemachatus* and Asian *Naja*) or with non-spitting cobras. Furthermore, given that the type and potency of anticoagulant venom effects varied greatly across the three spitting cobra lineages (Figure 3), this is suggestive of different selection pressures operating on the evolution of the venom arsenals of spitting cobras. Thus, it is unclear whether these potent anticoagulant actions have a predatory or defensive function in the venoms of the African spitting *Naja*.

Given the observed failure of SAIMR antivenom in this study to neutralise any of the anticoagulant effects of cobra venoms, which is consistent with clinical observations of antivenom ineffectiveness [19], we tested the neutralising potential of LY315920, an anti-inflammatory drug candidate originally designed as an inhibitor of mammalian PLA_2_s. As snake venom PLA_2_s play an important role in morbidity resulting from snakebite (e.g., tissue destruction, haemorrhage, haemolysis) [24], it is important to investigate the possibility of a broad-spectrum PLA_2_ inhibitor neutralising venom PLA_2_s. Our results revealed a promising ability of LY315920 to neutralise two different types of anticoagulant venom effects by cobra venoms: thrombin inhibition and inhibition of FXa’s ability to form a prothrombinase complex with FVa. This is consistent with previous work on other venom types which revealed that LY315920 and its variants are effective in preventing myotoxic and neurotoxic venom PLA_2_-driven toxicity [20,25,26,27]. This results in this study are, however, the first time that the ability of LY315920 to prevent potent coagulotoxic effects has been shown. Thus, while the antivenom failure observed in the present study is cause for concern, the effectiveness of LY315920 is extremely promising and should be the focus of follow-up in vitro and in vivo studies, and, ultimately, in clinical trials.

The data in this study revealed for the first time the ability of LY315920 to neutralise the anticoagulant effects caused by PLA_2_ toxins, thereby suggesting it as a promising candidate to treat cobra snakebites as a cheap and readily available drug that has been shown to be highly effective against a number of medically relevant, toxicological effects. LY315920 could potentially be used in combination with other small molecule inhibitors, thereby contributing to the development of affordable, broad-spectrum first-aid and clinical treatment. Future work must, however, investigate critical unanswered questions regarding the use of LY315920 in a clinical setting, including the efficacy of various administration routes (oral or injection, alone or in combination with antivenom). Similarly, the impact of these drugs’ potential suppression of endogenous PLA_2_-mediated physiological functions in the setting of snakebite needs to be ascertained. As the local damage caused by some snakebites could impede blood flow, oral or intravenous delivery of LY315920 may not be efficient if local damage has already occurred. Direct local injection may be the most efficient treatment plan for first aid or clinical therapy, and, thus, modes of delivery also should be investigated. Other questions include the possible need for additional therapeutic agents to achieve effective, broad-spectrum treatment, as well as determining optimal dosing schedules [20,25]. Extensive further research under lab conditions is therefore needed to validate the use of LY315920 prior to clinical trials and possible future clinical use.

A particularly intriguing phylogenetic trend is that the African spitting cobra clade we have documented in this study as being unusual for African and Asian elapids in having potent anticoagulant effects is also the same clade that is notorious for producing severe localised tissue destruction [19,28,29,30,31,32]. A recent study showed that most cobra species that use a hooding display in defense are more potently cytotoxic compared to non-hooding species, with cytotoxicity evolving on two separate occasions: once in the last common ancestor of *Hemachatus* and *Naja* (containing cyotoxic three-finger toxins in the venom) and independently in *Ophiophagus* (containing cytotoxic L-amino acid oxidases in the venom) [16]. Furthermore, a higher degree of these pain-inducing defensive toxins is exhibited by hooding species that are brightly coloured with strong aposematic markings [16]. In addition, high levels of defensive cytotoxins were also observed in the three independent lineages which have evolved spitting (i.e., spraying or squirting) of venom [16,33]. Thus, while African spitting *Naja* species are not significantly more cytotoxic than other spitting cobra lineages [16], they are unique in causing extreme local tissue damage to envenomed patients. Therefore, it seems likely that cytotoxicity alone does not account for the tremendous local tissue damage caused by bites of these species, but rather another component of the venom may be exacerbating the cytotoxic effects, perhaps in a synergistic manner. Whether there is a synergistic action with the anticoagulation noted in this study should be the focus of future research.

In summary, we determined the mechanism of anticoagulant venom activity and potency across all African and Asian elapid snake radiations, which provides additional insight into the pathophysiology of cobra snakebites. The revelation of potent anticoagulation in the African *Naja* species thus informs on evolutionary biology in addition to clinical medicine. Our study illustrates the importance of investigating under-studied venom effects, particularly nonlethal effects that may contribute to permanent disability.

## 4. Materials and Methods

The species studied were *Aspidelaps lubricus*, *A. scuttatus*, *Bungarus fasciatus*, *Dendroaspis polylepis*, *Elapsoidea boulengeri*, *E. sundevallii longicauda*, *E. s. sundevallii*, *Hemachatus haemachatus*, *Naja annulata*, *N. annulifera*, *N. atra*, *N. haje*, *N. kaouthia*, *N. melanoleuca*, *N. mossambica*, *N. naja* (India and Pakistan localities), *N. nigricincta*, *N. nigricollis*, *N. nivea*, *N. pallida*, *N. phillippinensis*, *N. samarensis*, *N. siamensis*, *N. sumatrana*, *Ophiophagus hannah* (Cambodia, Java, Malaysia, and Thailand localities), and *Walterinnesia aegyptia*. Venom samples were sourced from the long-term research collection of the Venom Evolution Lab, except *Naja nigricincta* and *N. samarensis* which were provided by FJV. Venoms were freeze-dried and stored at −80 °C until use. One-milligram aliquots of venom were reconstituted in deionised water and centrifuged (14,000 RCF at 4 °C for 10 min). The supernatant was then collected, and the protein concentration was measured using a ThermoFisher Scientific NanoDrop™ spectrophotometer. Working stocks of 1 mg/ml were made using 50% Milli-Q and 50% glycerol (>99% Sigma-Aldrich, St. Louis, MO, USA) and stored at −20 °C to reduce enzyme degradation. All venom work was undertaken under University of Queensland Biosafety Approval #IBC134BSBS2015.

### 4.1. Antivenom

The antivenom used for this assay was SAIMR Polyvalent Snake Antiserum/Antivenom from South African Vaccine Producers (Pty) Ltd., Johannesburg, 2192, South Africa. This antivenom was prepared against the venom of *Bitis arietans*, *Bitis gabonica*, *Dendroaspis polylepis*, *Hemachatus haemachatus*, and *Naja mossambica*. The antivenom was centrifuged at 14,000 RCF to remove any particulates, and the supernatant used for testing.

### 4.2. Human Plasma

Human plasma was obtained from the Australian Red Cross (research agreement #18-03QLD-09 and University of Queensland Human Ethics Committee Approval #2016000256). All plasma was prepared as 3.2% citrated stock, aliquoted into 1 ml quantities which were snap-frozen in liquid nitrogen, and stored in a −80 °C freezer until required, at which time an aliquot was defrosted in a 37 °C water bath for 5 min. All plasma work was undertaken under University of Queensland Biosafety Approval #IBC134BSBS2015.

### 4.3. Human Fibrinogen

Human fibrinogen was reconstituted to a concentration of 4 mg/mL in isotonic saline solution, flash-frozen in liquid nitrogen, and stored at −80 °C until use. When an aliquot was required, it was defrosted in a 37 °C water bath for 5 min.

### 4.4. Coagulation Assays Using the Stago STA-R Max

Plasma coagulation analyses and relative antivenom efficacy tests were performed using plasma clotting time as a proxy to assess the clotting capabilities of the various venoms (Table 1). The analyses were performed using a modified anticoagulant protocol on a Stago STA-R Max coagulation robot using Stago Analyser software v0.00.04 (Stago, Asnières sur Seine, France). Calcium and phospholipid were also added in order to imitate in vivo conditions present in the human body. Controls were performed using stocks of 50% Milli-Q and 50% glycerol as a replacement for venom to represent the spontaneous clotting time of human plasma under natural conditions.

Incubating a specific coagulation factor with the venom for 120 s allowed for the coagulotoxins to bind to and inhibit the targeted coagulation factor and exhibit the anticoagulant activity (Kini, 2006). All tests were undertaken as 8-point dilution curves of 20 μg/mL, 10 μg/mL, 4 μg/mL, 1.67 μg/mL, 0.67 μg/mL, 0.25 μg/mL, 0.125 μg/mL, and 0.1 μg/mL venom concentrations.

### 4.5. Phylogenetic Comparative Analyses

Coagulation times (seconds) for each of the venom and antivenom tests were graphed using Prism 7.0 software (GraphPad Software Inc, La Jolla, CA, USA) to produce concentration response curves. Data are expressed as mean ± SD for each triplicate data point. The phylogenetic tree used was based upon a previously published species tree [17] and created using Mesquite software (version 3.2), which was then imported into Rstudio using the APE package [34]. Ancestral states were estimated for all traits using the maximum likelihood as implemented in the contMap function of the R package phytools [35]. We then fit PGLS models [36] in caper [37] to test for relationships. As per previous studies using these methods [38,39], we used the phytools and PGLS scripts (Supplementary File S1).

## Figures and Tables

**Table 1:** Anticoagulation assays performed across all venoms.

Thrombin inhibition assay	Step 1. 25 µL of 0.2 mg/mL venom (1 mg/mL 50% glycerol stock diluted with Owren Koller (OK) Buffer (Stago Catalog # 00360) + 75 µL of [50 µL 0.025 M calcium (Stago catalog # 00367 + 25 µL of OK buffer] + 50 µL of phospholipid (Stago catalog #00597) + 25 µL of thrombin (Stago catalog # 00611). Step 2. 120 second incubation. Step 3. Addition of 75 µL of 4 mg/mL fibrinogen.
Thrombin inhibition assay (antivenom neutralisation tests)	Step 1. 25 µL of 0.2 mg/mL venom (1 mg/mL 50% glycerol stock diluted with OK Buffer + 75 µL of [50 µL 0.025 M calcium + 25 µL of 5% concentration antivenom] + 50 µL phospholipid + 25 µL of thrombin. Step 2. 120 s incubation. Step 3. Addition of 75 µL of 4 mg/mL fibrinogen.
Thrombin inhibition assay (LY315920 [varespladib] neutralisation tests)	Step 1. 25 µL of 0.2 mg/mL venom (1 mg/mL 50% glycerol stock diluted with OK Buffer + 75 µL of [50 µL 0.025 M calcium + 25 µL of 0.025 mg/mL LY315920] + 50 µL phospholipid + 25 µL of thrombin. Step 2. 120 s incubation. Step 3. Addition of 75 µL of 4 mg/mL fibrinogen.
FXa inhibition assay	Step 1. 25 µL 0.2 mg/mL venom (1 mg/mL 50% glycerol stock diluted with OK Buffer + 75 μl of [50 µL 0.025 M calcium +25 µL OK buffer] + 50 µL of phospholipid + 25 µL of FXa (Stago catalog # 00311). Step 2. 120 s incubation. Step 3. Addition of 75 µL plasma.
FXa inhibition assay (antivenom neutralisation tests)	Step 1. 25 µL 0.2 mg/mL of venom (1 mg/mL 50% glycerol stock diluted with OK Buffer + 75 µL of [50 µL 0.025 M calcium + 25 µL of 5% concentration antivenom] + 50 µL of phospholipid + 50 µL of FXa. Step 2. 120 s incubation. Step 3. Addition of 75 µL of plasma.
FXa inhibition assay (LY315920 neutralisation tests)	Step 1. 25 µL of 0.2 mg/mL venom (1 mg/mL 50% glycerol stock diluted with OK Buffer + 75 µL of [50 µL 0.025 M calcium + 25 µL of 0.025 mg/mL LY315920] + 50 µL of phospholipid + 25 µL of FXa. Step 2. 120 s incubation. Step 3. Addition of 75 µL of plasma.

## Supplementary Materials

The following are available online at http://www.mdpi.com/2072-6651/10/12/516/s1, Supplementary File S1: Phytools scripts.
